# Factors promoting research activities among Japanese pharmacists: a questionnaire survey

**DOI:** 10.1038/s41598-024-53454-w

**Published:** 2024-02-05

**Authors:** Masaki Takigawa, Yuki Kondo, Yutaka Kobayashi, Akane Iihoshi, Masako Kinoshita, Yoichi Ishitsuka, Masayuki Masuda

**Affiliations:** 1 Department of Clinical Pharmaceutics, Faculty of Pharmaceutical Sciences, Toho University, 2-2-1 Miyama, Funabashi, Chiba 274-8510 Japan; 2 Department of Clinical Chemistry and Informatics, Graduate School of Pharmaceutical Sciences, Kumamoto University, 5-1 Oe-Honmachi, Chuo-Ku, Kumamoto, 862-0973 Japan; 3 Department of Pharmacy, Fujinomiya City General Hospital, 3-1 Nishiki-Cho, Fujinomiya, Shizuoka 418-0076 Japan; 4 Department of Pharmacy, Seirei Yokohama Hospital, 215, Hodogaya, Yokohama, Kanagawa 240-8521 Japan

**Keywords:** Medical research, Health care

## Abstract

Pharmacists are expected to demonstrate their expertise in clinical practice and conduct research activities to generate new evidence. However, the factors promoting research activities among pharmacists remain unclear. Therefore, we investigated the research activities of Japanese pharmacists through a questionnaire survey and examined the factors contributing to the promotion of research activities. A web-based questionnaire using Google Forms was disseminated across pharmacists working in community pharmacies, drugstores, hospitals, and clinics. The questionnaire included respondents’ backgrounds, research activities, and research environments. Logistic regression analysis was used to examine the factors promoting pharmacists’ research activities, with experience in research paper acceptance as the objective variable. In total, 401 responses were included in the analysis. Of the respondents, 54.1% were hospital pharmacists, and 77.1% were pharmacists with > 5 years of pharmacist experience. Furthermore, 50.4% of the pharmacists had presented at conferences, and 22.2% had experience in research paper acceptance. The influential factors were “master’s degree or higher,” “number of affiliated academic societies,” “acquisition of specialists/certified pharmacists,” and “daily availability of a consultant for writing research papers.” This study revealed the factors contributing to the promotion of research activities among pharmacists. We believe that our findings will help promote research among pharmacists.

## Introduction

Pharmacists are expected to demonstrate clinical expertise and conduct research to generate new evidence. In the United States, residency programs focus on clinical training, whereas fellowship programs focus on research. Earlier, it was necessary to graduate from a four-year faculty of pharmacy to become a pharmacist in Japan. However, a six-year pharmacy school system was introduced in 2006, and now students must graduate from a six-year faculty of pharmacy to become a pharmacist. The purpose of this two-year extension of study is to develop diverse human resources for drug development and drug-related research, in addition to enhancing clinical education. To encourage the development of research skills, pharmaceutical research is included in Japan’s model core curriculum for pharmaceutical education. In addition, numerous efforts are being made to promote pharmacists’ research activities^1–3^. In Japan, practicing pharmacists have to undertake research activities to obtain some of the specialist pharmacist certifications granted by various societies. For example, pharmacists require "more than 2 presentations at conferences" and "at least one research article as the first author to become a "Board certified Clinical Pharmacist-Scientist", a certification granted by the Japanese Society of Pharmaceutical Health Care and Sciences. The acquisition of such a specialty pharmacist is an opportunity to provide specialized knowledge in a clinical setting. In addition, a pharmacist's research activities contribute to career advancement. For example, research accomplishments and degrees may be required for pharmacists to become managers in the pharmacy department of the hospital.

Several studies have been conducted on pharmacists’ research activities. Watanabe et al.^4^ surveyed hospital pharmacists regarding their research interests. Koutake et al.^5^ surveyed hospital pharmacists regarding their clinical research experience; 48.2% of hospital pharmacists had presented at a conference, and 15.4% had published a research paper. In addition, approximately 80% of the respondents believed that research by pharmacists was necessary, and only 21.4% of pharmacists were conducting research at the time of the survey, indicating that research activities were not being conducted sufficiently to meet the needs^5^.

The factors that stimulate research activities among healthcare professionals have also been reported. “Mentorship” and “Protected for scholarly or administrative duties” drive academic activity among U.S. hospitalists^6^. According to a survey, “Time to perform research during working hours” and “Regular research conferences” are associated with academic activities^7^. Thus, factors that promote research activities among non-pharmacist healthcare professionals have been identified. However, the factors promoting research activities among pharmacists remain unclear.

Therefore, this study conducted a questionnaire survey to investigate the research activities and research environment of pharmacists from hospitals and pharmacies and examine factors contributing to promoting pharmacists’ research activities.

## Results

### Respondents' characteristics

The characteristics of the survey respondents are listed in Table 1. In total, 404 pharmacists agreed to participate in the survey. Of these, 401 responses were included in the analysis, excluding three responses that contained invalid responses. There were 223 male respondents (55.6%) and 178 female respondents (44.4%). Of the respondents, 313 (78.1%) had a bachelor’s degree, 92 (22.9%) had < 5 years of experience as a pharmacist, and 302 (77.1%) had > 5 years of experience. Of the respondents, 217 (54.1%) were hospital pharmacists, and 184 (45.9%) were community pharmacists. Of the respondents, 117 (29.2%) had certifications or were professional pharmacists who required research activity.

**Table 1:** Respondent characteristics.

Characteristic	No. of patients (%) or Mean (Range)
Sex	
Male	223 (55.6)
Female	178 (44.4)
Undergraduate curriculum	
4-years degree	196 (48.9)
6-years degree	205 (51.1)
Educational background	
Bachelor’s degree	313 (78.1)
Master’s degree	52 (13.0)
Doctorate degree	36 (9.0)
Work experience as a pharmacist	
5 < years	92 (22.9)
5–9 years	94 (23.4)
10–19 years	134 (33.4)
> 20 years	81 (20.2)
Work place	
Hospital or clinic	n = 217
100 < beds	15 (6.9)
100–199 beds	16 (7.4)
200–299 beds	31 (14.3)
300–399 beds	40 (18.4)
400–499 beds	31 (14.3)
> 500 beds	84 (38.7)
Community pharmacy or drug store	n = 184
1–9 stores	66 (35.9)
10–19 stores	19 (10.3)
20–49 stores	16 (8.7)
50–99 stores	9 (4.9)
> 100 stores	74 (40.2)
Number of affiliated academic societies	1 (0–11)
Acquisition of specialist/certified pharmacist	117 (29.2)
Frequency of participation in academic conferences	
Participate at least once annually	211 (52.6)
Rarely or only once every few years	190 (47.4)

”Community pharmacy" refers to a pharmacy that deals with prescription medicines and "drug store" refers to a pharmacy that does not deal with prescription medicines.

## Research activities of respondents

The respondents’ research activities are listed in Table 2. Of the respondents, 202 (50.4%) had presented at academic conferences. Of the 217 hospital pharmacists, 152 (70.0%) had presented at academic conferences, and of the 184 community pharmacists, 50 (27.2%) had presented at conferences. Research papers of 89 respondents (22.2%) were accepted. Furthermore, research papers of 71 (32.7%) hospital pharmacists and 18 (9.8%) community pharmacists were accepted.

**Table 2:** Research activities of the respondents.

Questionnaire items	Total n, (%)	Hospital pharmacist, n (%)	Community pharmacists, n (%)
Experience in presenting at academic conferences	202 (50.4)	152 (70.0)	50 (27.2)
Experience in research paper acceptance	89 (22.2)	71 (32.7)	18 (9.8)

### Factors related to the presence and absence of accepted research papers

The univariate analysis results, with the presence and absence of experience in research paper acceptance as the objective variable, are presented in Table 3. Compared to the group with no experience in research paper acceptance, the group with experience in research paper acceptance had a higher percentage of respondents with a master’s degree or higher as their final educational background. The group with experience in research paper acceptance was affiliated with 3 (0–11) academic societies, whereas the group without experience in research paper acceptance was affiliated with 0 (0–8) academic societies. Furthermore, the group with experience in research paper acceptance included a higher percentage of respondents who acquired specialists/certified pharmacists and who attended academic conferences every year. In addition, most respondents from the group with experience in research paper acceptance had someone for daily consultations regarding presentations at academic conferences and writing research papers compared to the group without experience in research paper acceptance. Furthermore, a higher percentage of respondents in the group with experience in research paper acceptance had financial support for their academic activities and could obtain academic papers. The final logistic regression model identified that the factors associated with pharmacists’ research activities were “master’s degree or higher” (odds ratio [OR]: 7.00, 95% confidence interval [CI]: 3.44–14.24), “number of affiliated academic societies” (OR: 1.44, 95% CI: 1.18–1.75), “acquisition of specialists/certified pharmacists” (OR: 4.12, 95% CI: 2.06–8.26), and “daily availability of a consultant for writing research papers” (OR: 6.84, 95% CI: 3.46–13.53) (Table 4). The final model’s area under the receiver operating characteristics curve was 0.904.

**Table 3:** Comparison of groups with and without experience in research paper acceptance.

Questionnaire items	Experience in accepting research papers		*P*-value
	With, n (%) or Mean (Range)	Without, n (%) or Mean (Range)	
Sex			
Male	72 (80.9)	151 (48.4)	< 0.001^a^
Female	17 (19.1)	161 (51.6)	
Undergraduate curriculum			
4-years degree	55 (61.8)	141 (45.2)	0.006^a^
6-years degree	34 (38.2)	171 (54.8)	
Educational background			
Bachelor’s degree	38 (42.7)	275 (88.1)	< 0.001^a^
Master’s degree or higher	51 (57.3)	37 (11.9)	
Work experience as a pharmacist			< 0.001^a^
5 < years	8 (9.0)	84 (26.9)	
> 5 years	81 (91.0)	228 (73.1)	
Workplace			
Hospital or clinic	71 (79.8)	146 (46.8)	< 0.001^a^
Pharmacy or drug store	18 (20.2)	166 (53.2)	
Workplace size			
Hospital or clinic			
100 < beds	6 (8.5)	9 (6.2)	0.044^a^
100–199 beds	2 (2.8)	14 (9.6)	
200–299 beds	8 (11.3)	23 (15.8)	
300–399 beds	11 (15.5)	29 (19.9)	
400–499 beds	9 (12.7)	22 (15.1)	
>500 beds	35 (49.3)	49 (33.6)	
Community pharmacy or drug store			
1–9 stores	10 (55.6)	56 (33.7)	0.055^a^
10–19 stores	2 (11.1)	17 (10.2)	
20–49 stores	1 (5.6)	15 (9.0)	
50–99 stores	1 (5.6)	8 (4.8)	
> 100 stores	4 (22.2)	70 (42.2)	
Number of affiliated academic societies	3 (0–11)	0 (0–8)	< 0.001^b^
Acquisition of specialist/certified pharmacist	62 (69.7)	55 (17.6)	< 0.001^a^
Participation in academic conference			
Participate at least once annually	78 (87.6)	133 (42.6)	< 0.001^a^
Rarely or only once every few years	11 (12.4)	179 (57.4)	
Daily availability of a consultant for presenting at academic conferences	68 (76.4)	105 (33.7)	< 0.001^a^
Daily availability of a consultant for writing research papers	62 (69.7)	71 (22.8)	< 0.001^a^
Duty to present at academic conferences at the workplace	11 (12.4)	13 (4.2)	0.004^a^
Duty of publishing research papers at the workplace	8 (9.0)	4 (1.3)	< 0.001^a^
Financial support for academic conference travel and research paper submission	62 (69.7)	134 (42.9)	< 0.001^a^
Availability of academic papers	57 (64.0)	80 (25.6)	< 0.001^a^
Research day or research activities allowed during work hours	11 (12.4)	19 (6.1)	0.047^a^

^a^Chi-square test, ^b^Mann–Whitney U test. ”Community pharmacy" refers to a pharmacy that deals with prescription medicines and "drug store" refers to a pharmacy that does not deal with prescription medicines.

**Table 4:** Factors related to the experience in research paper acceptance.

Explanatory variable	Odd ratio	95% CI	*P*-value
Master’s degree or higher	7.00	3.44–14.24	< 0.001
Daily availability of a consultant for writing research papers	6.84	3.46–13.53	< 0.001
Acquisition of specialist/certified pharmacist	4.12	2.06–8.26	< 0.001
Number of affiliated academic societies	1.44	1.18–1.75	< 0.001

### Support for research activities

Table 5 lists the support needed for research activities for those who had never presented at a conference or published research papers and who had presented at a conference but had never published research papers. The most common response was "availability of a consultant and guide for research" with 150 responses from those who had never presented at a conference or published a research paper and 105 responses from those who had presented at a conference but had never published a research paper. The second most frequent response was "research days and research activities are possible during working hours," with 134 responses from those who had never presented at a conference or published a research paper and 81 responses from those who had presented at a conference but had never published a research paper.

**Table 5:** Support required for research activities by pharmacists with no experience in research paper acceptance.

Support for research activities	Number
A	
Availability of a consultant and guide for research	150
Joint research with universities and other institutions	59
Research activities are reflected in personnel appraisals in the workplace	63
Statistical software available	63
Financial support for academic conference travel and research paper submission	98
Availability of academic papers	82
Research day or research activities allowed during work hours	134
Unknown support needed for research	28
Other	7
B	
Availability of a consultant and guide for research	105
Joint research with universities and other institutions	51
Research activities are reflected in personnel appraisals in the workplace	47
Statistical software available	59
Financial support for academic conference travel and research paper submission	61
Availability of academic papers	57
Research day or research activities allowed during work hours	81
Unknown support needed for research	6
Other	8

The results are shown for (A) those who had never presented at a conference and published a research paper (n = 192, multiple answers allowed) and (B) those who had presented at a conference but had never published a paper (n = 120, multiple answers allowed).

## Discussion

In this study, pharmacists from community pharmacies, drugstores, hospitals, and clinics were surveyed using a questionnaire to investigate their research activities and backgrounds and identify factors that promote research activities. The factors related to the experience in research paper acceptance were “master’s degree or higher,” “number of affiliated academic societies,” “acquisition of specialist/certified pharmacist,” and “daily assistance for writing research papers.” The most common type of assistance needed by those without experience in research paper acceptance was the “availability of a consultant and guide for research.”

The number of responses received for this study was 401, which was higher than the calculated required sample size of 384. To examine whether the respondents in this study were consistent with the pharmacist composition in Japan, the background of this study participants was compared with the “Survey of Physicians, Dentists and Pharmacists in 2020^8^.” This survey showed that the total number of pharmacists in Japan was 321,982, with 188,982 pharmacists working in pharmacies and the 61,603 pharmacists working in medical facilities^8^. The study included 55.6% males and 44.4% females compared to 38.6% males and 61.4% females in the “Survey of Physicians, Dentists and Pharmacists in 2020.” In addition, 45.9% of respondents in this study were community pharmacists, and 54.1% were hospital pharmacists, compared to 58.7% of community pharmacists and 17.4% of hospital workers in the “Survey of Physicians, Dentists and Pharmacists in 2020.” Furthermore, 46.4% of the respondents in this study had < 5 or 5–9 years of experience as pharmacists. In contrast, the “Survey of Physicians, Dentists and Pharmacists in 2020” reported that 12.4% of the total respondents were 20–29 years old with < 10 years of experience as pharmacists. These results indicate that most respondents in this study were male, hospital pharmacists, and younger than the actual composition of pharmacists in Japan. The large percentage of younger respondents may have been because the survey was disseminated through social networking services (SNS), resulting in a higher percentage of younger respondents using SNS frequently. A higher percentage of hospital pharmacists in this survey reported having experience in both conference presentations and research paper writing compared to community pharmacists (Table 2). A higher percentage of hospital pharmacists may have responded to this survey because many hospital pharmacists interested in research responded to this survey.

This survey showed that 50.4% of the pharmacists had presented at conferences, whereas 22.2% of the pharmacists had experience in research paper acceptance. Although the survey did not target only pharmacists, Komagamine et al.^9^ reported that 3.8% of the abstracts from the Japan Primary Care Association Annual Meetings (2010–2012) were published as research papers. Furthermore, 19.8% of conference abstracts of pharmacists were published in research papers or other publications^10^. Thus, there may be obstacles between presenting at conferences and writing research papers. “Lack of time” has been reported as a reason conference abstracts have not been published^11^. In this study, most pharmacists without experience with research paper acceptance also cited “research day or research activities allowed during work hours” as a necessary support for their research activities. Although pharmacists experience difficulty writing research papers due to work hours, the percentage of pharmacists with experience in research paper acceptance may increase with sufficient available time. In this study, 70.0% of hospital pharmacists had presented at conferences, and 32.7% had experience in research paper acceptance. Koutake et al.^5^ reported that 48.2% of hospital pharmacists had presented at conferences, and 15.4% had published research papers. Wachi et al.^12^ reported that 74.4% of hospital pharmacists had presented at conferences, and 20.2% had contributed to research papers. Although the backgrounds of the study respondents and those from previous reports^5,12^ were different, a larger proportion of hospital pharmacists in this study had experience in research paper acceptance compared to those in the previous studies. There has been a report on research activities among community pharmacists in the UK and Australia^13^. Matsumoto et al.^14^ surveyed the awareness of evidence generation among community pharmacists in Japan. Matsumoto et al.^14^ reported that 16.8% of community pharmacists had published research papers as students and 6.8% had published papers after graduation. In the present study, 9.8% of the community pharmacists had experience accepting research papers, which is generally consistent with the report by Matsumoto et al.

According to the final logistic regression model, the most influential factors were “master’s degree or higher,” “number of affiliated academic societies,” “acquisition of specialist/certified pharmacist,” and “daily availability of a consultant for writing research papers.” “master’s degree or higher” had the highest odds ratio may be because pharmacists with a master’s or doctoral degree have experience in writing research papers, and those interested in research pursue a master’s degree or higher. “acquisition of specialist/certified pharmacist” may also be correlated with research activities conducted to fulfill certification requirements. “Daily availability of a consultant for writing research papers” had the second-highest odds ratio. A previous study also reported that the “availability of a mentor” is necessary for research activities, which is consistent with the results of this study^6^. The respondents’ “Number of affiliated academic societies” was also correlated with experience in research paper acceptance. Pharmacists affiliated with many academic societies actively collect information and participate in academic activities, which may be related to their experience in research paper acceptance.

Respondents with no experience in research paper acceptance (those with no experience of presenting at conferences and research paper acceptance and those who had presented at conferences but had never published research papers) were surveyed to determine the support they needed for their research activities. The most necessary support for both groups was the “availability of a consultant and guide for research.” The second most required support for both groups was “research day or research activities allowed during work hours,” which was related to time for research. Another survey reported “availability of a mentor” and “fixed time for research” as necessary for research activities by physicians, consistent with the support needed for research activities by pharmacists^6,7^. The logistic regression analysis results revealed that “daily availability of a consultant for writing research papers” was associated with experience in research paper acceptance. Therefore, developing a guidance system for writing research papers may be useful for pharmacists with no experience in research paper acceptance.

Currently, the number of students in Japan who go on to join doctoral programs after graduation from the school of pharmacy is decreasing. As a solution to this problem, we think it may be useful to encourage pharmacists to enroll in doctoral programs. Pharmacists can obtain the guidance of research supervisors by joining doctoral programs. In addition, seeing pharmacists conducting research may encourage pharmacy students to consider the option of advancing to a doctoral program after graduation. Such efforts could also lead to the promotion of research activities by pharmacists.

This study had several limitations. First, because this was a web-based survey, the respondents may be biased toward pharmacists interested in research activities^15^. Furthermore, pharmacists who are not engaged in research activities may feel stigmatized by this survey^16^. Even pharmacists who are not engaged in research activities were likely to respond to the web surveys. We conducted a web-based survey to gather the opinions of numerous pharmacists. In addition, since this survey used Google Forms, it may contain duplicate responses from the same respondent. Although we checked for multiple entries by respondents as much as possible based on their backgrounds, it is possible that we may not have eliminated all duplicate responses. Second, most respondents in this study were male, hospital pharmacists, with an average age younger than that of pharmacists according to Japanese national statistics. Therefore, this may not accurately reflect the current situation of pharmacist research activities in Japan. In addition, the results of this study may have been obtained from a biased population. Third, in this study, it was not explicitly stated that academic conference proceedings from presentations do not qualify as research papers. However, in Japan, where many conferences do not undergo peer review during presentations, it is likely that most participants distinguish between the two clearly.

In conclusion, these results suggest the advantage of promoting research activities among pharmacists by providing understanding and support in the workplace and establishing a system for consultation on research activities by academic societies. We believe that the results of this study provide useful information to further promote the research activities of pharmacists.

## Methods

### Study design

A web-based questionnaire created using “Google Forms” was distributed to pharmacists in community pharmacies, drugstores, hospitals, and clinics. The URL to access the questionnaire was widely disseminated through SNS to encourage participation in the survey and avoid bias. The main SNS used to announce the questionnaire was X, formerly Twitter. The survey results were tallied from September 01 to October 31, 2022. A description of the questionnaire content was displayed to the survey respondents. After that, informed consent was obtained from all participants by having them select "yes" to the question "Do you agree to answer the questionnaire?”. The questionnaire inquired regarding respondents’ background (sex, undergraduate curriculum, educational background, years of experience as a pharmacist, workplace, number of affiliated academic societies, acquisition of specialist/certified pharmacists, and frequency of participation in academic conferences), research activities (whether and how frequently they presented at academic conferences and whether their research papers were accepted), and research environment (availability of mentors for research activities, availability of research papers, financial support for research activities, and availability of research days). The research paper submission was the final output of the research results. Therefore, respondents without accepted research papers were investigated for the support needed for their research activities.

### Statistical analyses

In 2020, the number of pharmacists in Japan was 321,982^8^. For this population, the required sample size was calculated to be n = 384, assuming a 50% collection rate for each facility, a 95% confidence level, and a 5% margin of error.

The responses of individuals actively engaged in research activities were compared with those not engaged in research activities to examine the factors contributing to the promotion of research activities among pharmacists based on these differences. Herein, a pharmacist actively engaged in research is someone with “experience of being accepted as the first author in peer-reviewed articles.” The reason for choosing the first author instead of the corresponding author is that we wanted to investigate the background of pharmacists who can accomplish the submitting a research paper, not just end up presenting it at an academic conference. Logistic regression analysis was performed with the presence or absence of experience in accepted research papers as the objective variable. Explanatory variables were variables with *P*-values < 0.1 in univariate analysis. Univariate analysis was performed to compare the groups with and without individuals with accepted research papers using the chi-square test or the Mann–Whitney U test. Bivariate analysis was performed to confirm multicollinearity, and when a strong correlation was found, such that |r|> 0.8, a variable with a small *P*-value was inserted. Logistic regression was performed using stepwise model selection (forward stepwise selection) according to the Bayesian information criterion. All statistical analyses were performed using JMP Pro 17 (SAS Institute Inc., Cary, NC, USA).

### Ethical approval

This study was approved by Tokyo Metropolitan Geriatric Hospital’s ethics committees after it was confirmed that it does not fall under the "Ethical Guidelines for Medical and Biological Research Involving Human Subjects" (approval no. R21-112). The study was also conducted in accordance with the latest version of the Declaration of Helsinki.

## Data Availability

Data are available from the corresponding author upon reasonable request.
